# Supplementary figures and images for: Human exposure to PM10 microplastics in indoor air
Source: PLoS One. 2025 Jul 30;20(7):e0328011. doi: 10.1371/journal.pone.0328011 (PMC12310009; doi:10.1371/journal.pone.0328011)

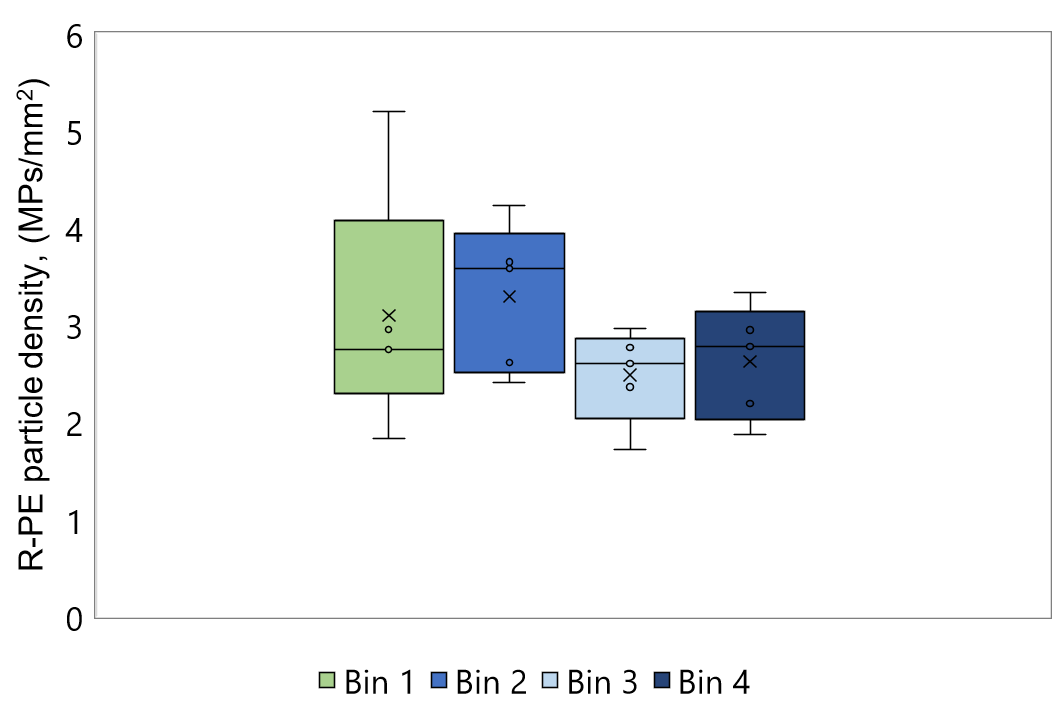

Supplement: S1 Fig — Bins 1–4 correspond to concentric rings with mean distances from the center at 1.25, 3.75, 6.25, and 8.5 mm (S3 Table). (TIF) [file pone.0328011.s005.tif]

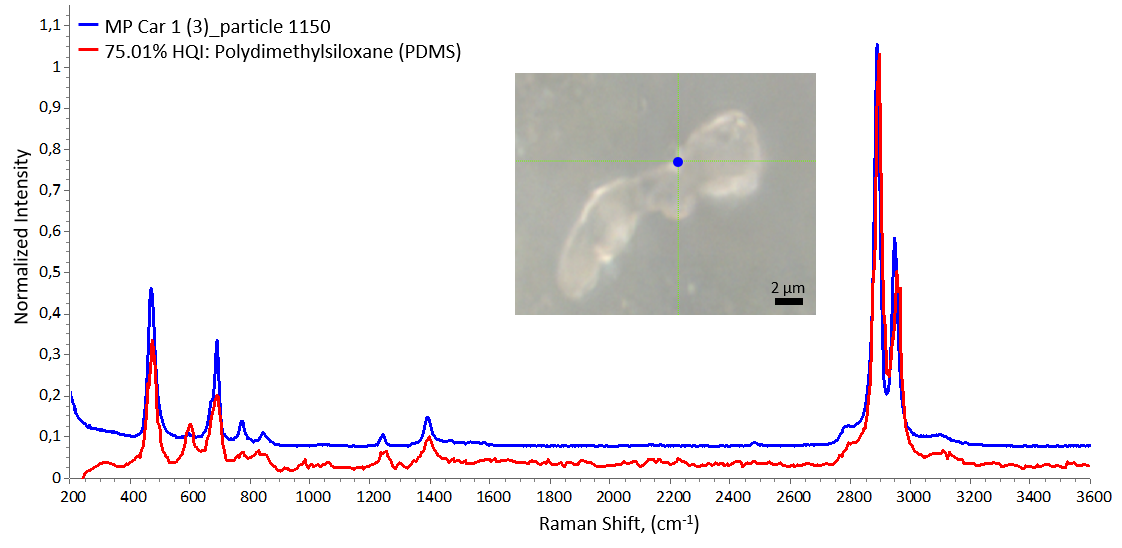

Supplement: S2 Fig — (TIF) [file pone.0328011.s006.tif]

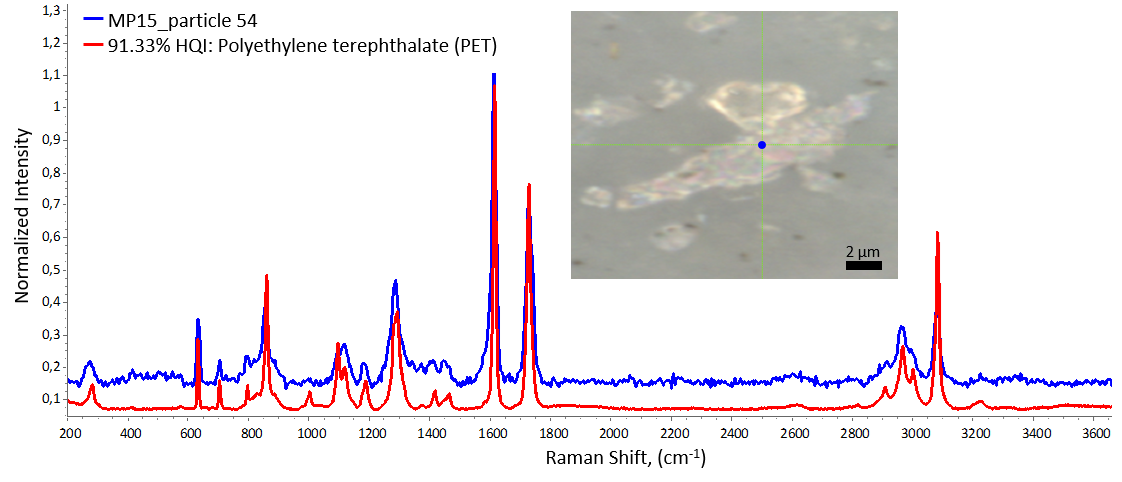

Supplement: S3 Fig — (TIF) [file pone.0328011.s007.tif]

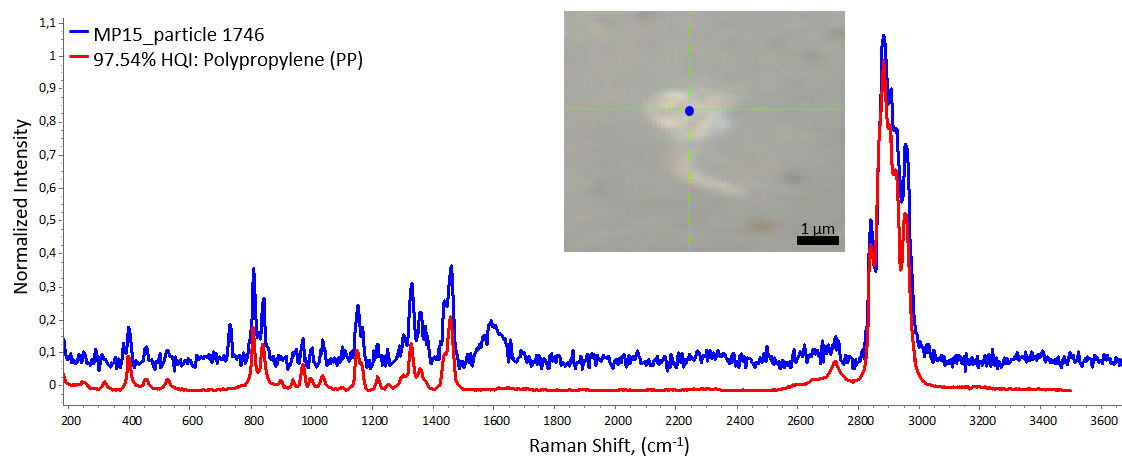

Supplement: S4 Fig — (TIF) [file pone.0328011.s008.tif]

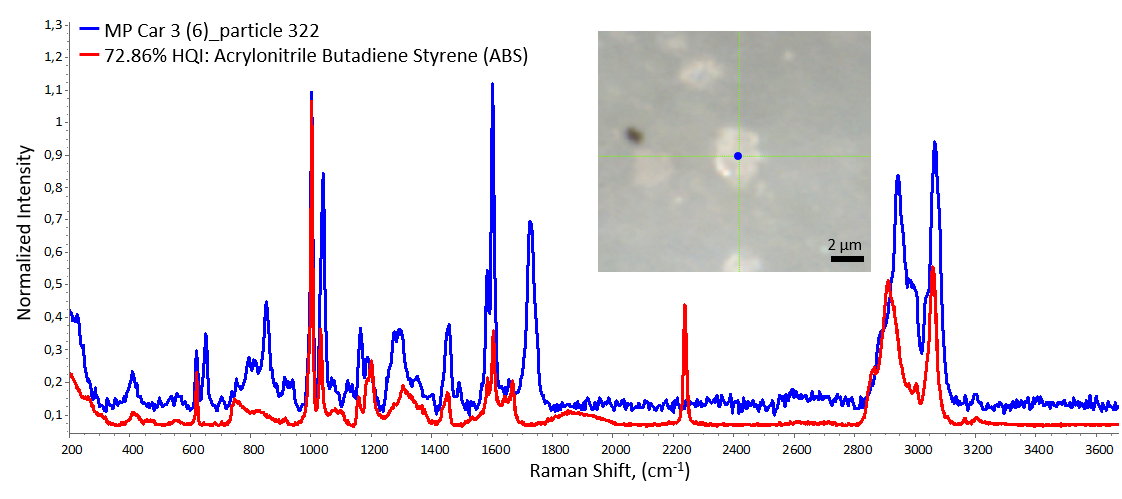

Supplement: S5 Fig — (TIF) [file pone.0328011.s009.tif]

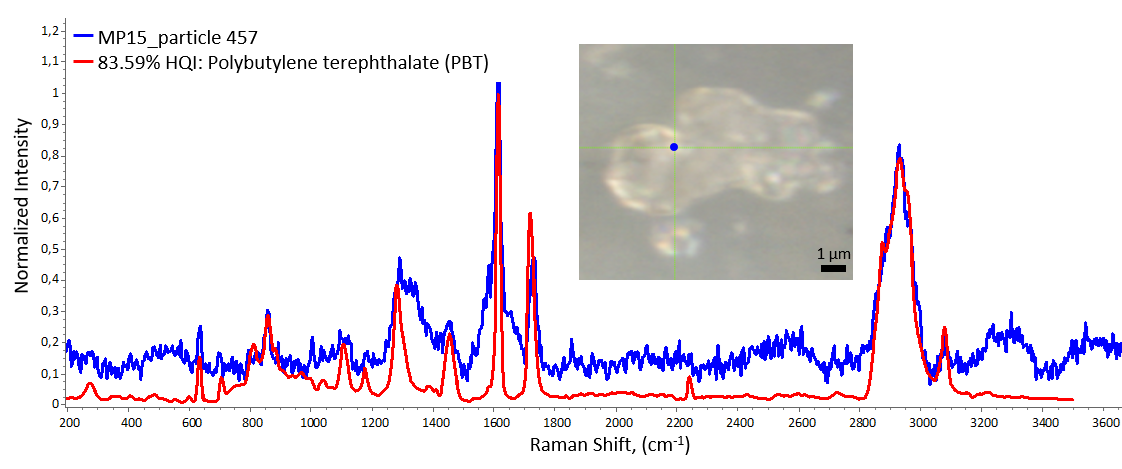

Supplement: S6 Fig — (TIF) [file pone.0328011.s010.tif]

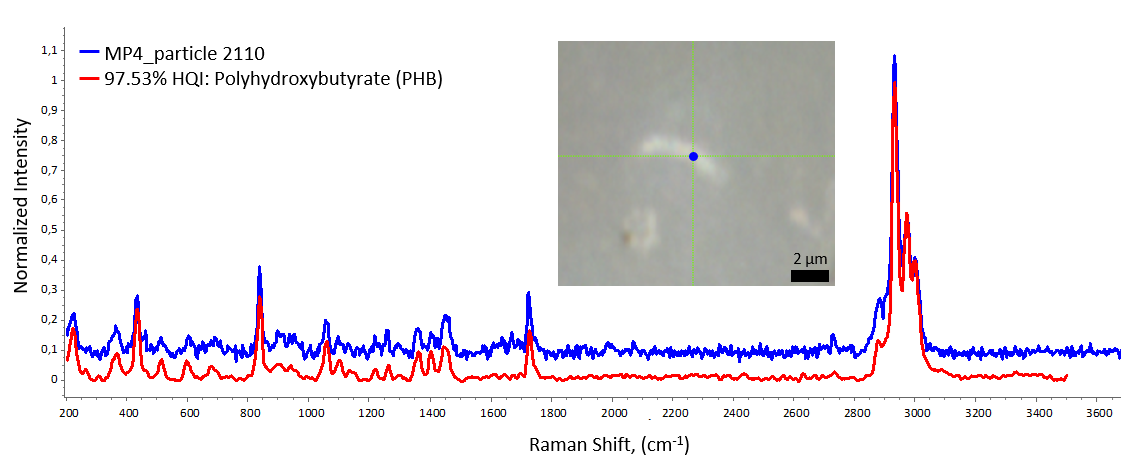

Supplement: S7 Fig — (TIF) [file pone.0328011.s011.tif]

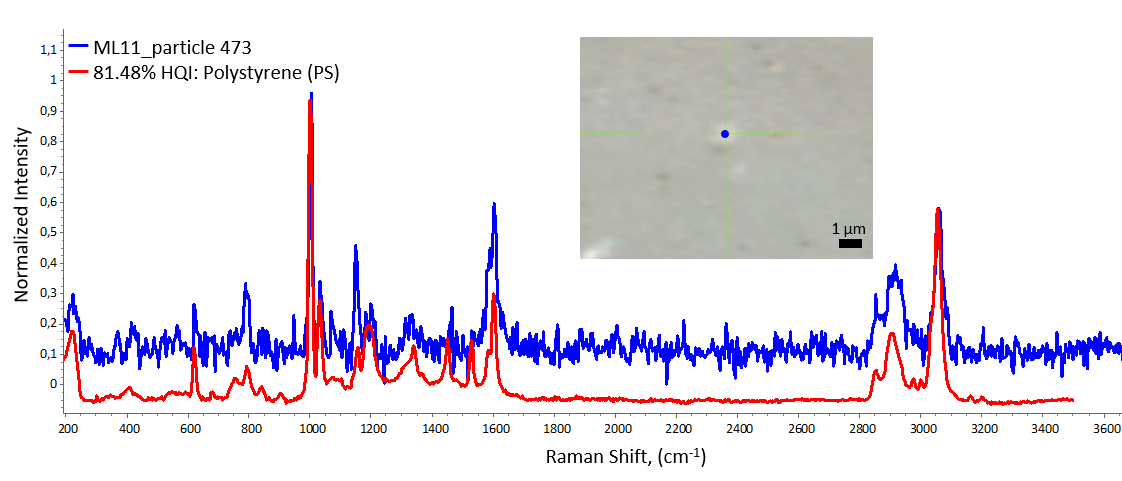

Supplement: S8 Fig — (TIF) [file pone.0328011.s012.tif]

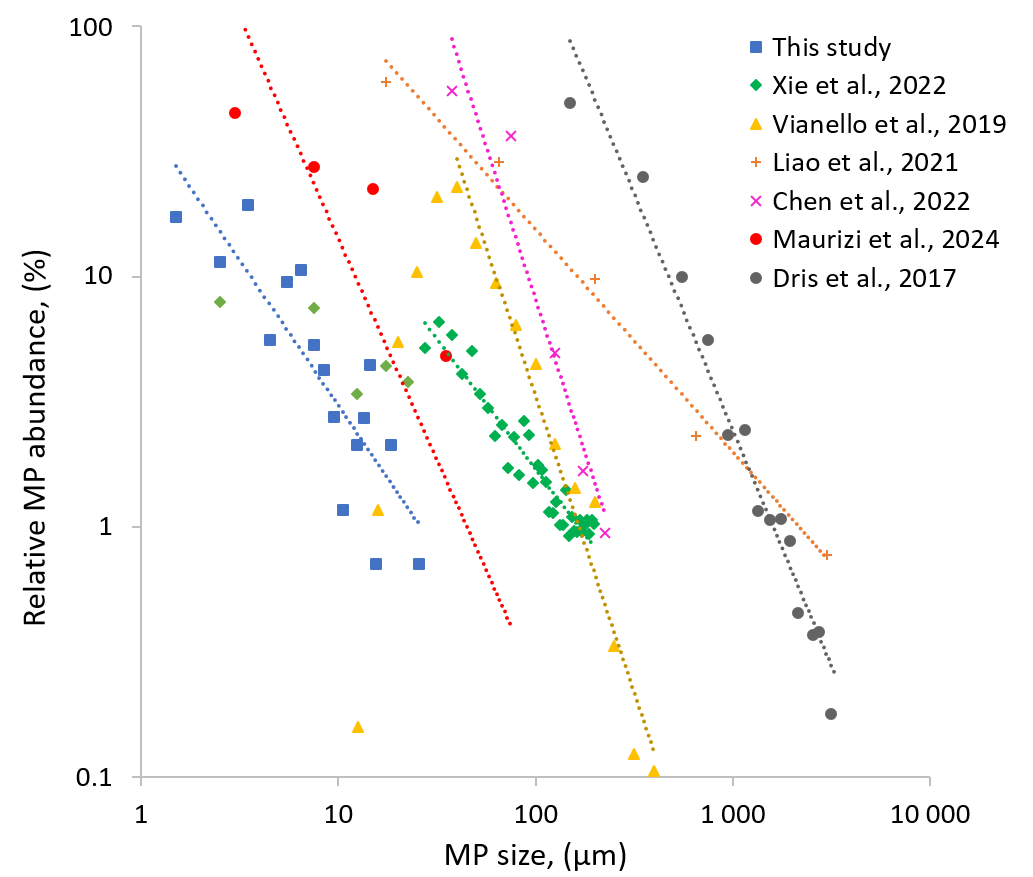

Supplement: S9 Fig — Power law distributions are fitted for each study: y = bx–α and have a mean exponent α of −1.65 ± 0.63(1σ). (TIF) [file pone.0328011.s013.tif]
