# Supplementary material for: Human exposure to PM10 microplastics in indoor air
Source: PLoS One. 2025 Jul 30;20(7):e0328011. doi: 10.1371/journal.pone.0328011 (PMC12310009; doi:10.1371/journal.pone.0328011)
Supplement: Attachment — Submitted filename: REVIEW plos one.pdf [file pone.0328011.s014.pdf]

REVIEW:

This manuscript is an interesting and important contribution to the study of airborne microplastics. The topic is highly relevant, and the findings add valuable insights into indoor exposure, which makes it even more compelling. The study is well thought out, and the methods are strong.

The comments below are just suggestions to help improve clarity and readability. The research is already solid, and with a few changes, it will be even stronger and easier for readers to engage with.

1) **Lines 24–26:** The abstract presents interesting findings, but the take-home message could be clearer. Consider emphasizing why your study is particularly significant compared to previous work. For example, rephrase the last two sentences to highlight the critical health implications more directly.

2) **Line 20:** The term "power size distribution law" is mentioned in the abstract but not explained. Since this may not be familiar to all readers, a brief clarification (even a parenthetical note) would improve accessibility.

3) **Lines 72–78**: The inclusion of the car cabin environment is an interesting and novel aspect, but its significance isn't immediately clear. Would it be useful to briefly explain why car environments were chosen alongside residential space? perhaps highlighting their relevance to daily exposure?

4) **Lines 62–65:** The introduction covers a lot of ground effectively, but the transition to the research gap could be smoother. The sentence starting with "However, despite its potential, only two studies [27,28]..." feels abrupt. Could you introduce this section with a more gradual lead-in?

5) **Lines 14, 20, 68 .. :** The manuscript switches between "MP1–10μm" and "MP1–10 μm" (with and without a space). Standardizing this across the text will improve readability.

6) **Lines 88–90:** The choice of sampling heights (1.6–1.7 m for living rooms and 0.5 m for bedrooms) makes sense intuitively but would benefit from a brief justification. Does this reflect average breathing heights?

7) **Lines 188–192:** the author mentions that blank corrections were applied, but it would be helpful to explain if any contamination patterns were observed across different polymer types. Did specific polymers appear more frequently in blanks, and if so, how was this addressed?

8) **Lines 320–322:** the author reports that MP concentrations were higher in cars than in apartments but also mention that this difference was not statistically significant ($p = 0.5$). Perhaps rewording to avoid implying a strong contrast would prevent misinterpretation.

9) **Lines 366–369:** The estimated inhalation rates are striking, but a brief discussion of uncertainty sources (e.g., variability in human breathing rates, exposure conditions) would help contextualize the robustness of these values.

10) **Lines 272–276, 300–301:** The figures (1,2,3 and 4) are informative, but are the labels and axis text large enough for clear readability? I suggest improving the image quality by using higher-resolution versions.

11) **Lines 317–319:** The discussion highlights how your estimates are higher than previous extrapolations, which is great. However, a sentence explaining why earlier estimates were lower (e.g., due to detection limits, methodology differences) would provide helpful context.

12) **Lines 348–350:** The study focuses on indoor exposure, but some readers may wonder how these findings compare to occupational environments with high plastic use (e.g., textile industries). A brief mention of this in the discussion could add depth.

13) **Lines 381–384:** The potential health impacts of inhaling MP1–10µm are mentioned, but could you add one or two specific examples of known biological effects (e.g., inflammation, oxidative stress) to make this more concrete?

14) **Lines 395–398:** The conclusion does a great job summarizing findings, but a forward-looking statement about next steps in this field (e.g., improving detection methods, longitudinal exposure studies) would make for a stronger closing.
