# Supplementary material for: Human exposure to PM10 microplastics in indoor air
Source: PLoS One. 2025 Jul 30;20(7):e0328011. doi: 10.1371/journal.pone.0328011 (PMC12310009; doi:10.1371/journal.pone.0328011)
Supplement: Attachment — Submitted filename: Response to Reviewers.docx [file pone.0328011.s017.docx]

**Nadiia Yakovenko and Jeroen E. Sonke**

CNRS & Université de Toulouse

Géosciences Environnement Toulouse

14 Av. Edouard Belin, 31400 Toulouse, France

**5 June 2025**

**Rebuttal letter**

**Manuscript:** PONE-D-25-08822 “Human exposure to PM_10_ microplastics in indoor air”

We thank the reviewers and editor for their constructive comments and questions. Below, point by point replies are given in red, and modifications (added text) in blue.

**Journal requirements:**

When submitting your revision, we need you to address these additional requirements.

**1.** Please ensure that your manuscript meets PLOS ONE's style requirements, including those for file naming. The PLOS ONE style templates can be found at

https://journals.plos.org/plosone/s/file?id=wjVg/PLOSOne_formatting_sample_main_body.pdf and

https://journals.plos.org/plosone/s/file?id=ba62/PLOSOne_formatting_sample_title_authors_affiliations.pdf

**2.** In your Methods section, please provide additional information regarding the permits you obtained for the work. Please ensure you have included the full name of the authority that approved the field site access and, if no permits were required, a brief statement explaining why.

We have added clarification to the Materials and Methods section on Line 85 (L85): “No permits were required because the residential and car environments studied are all privately owned by the authors of this study.”

**3.** Thank you for stating the following financial disclosure:

[ANR-20-CE34-0014 ATMO-PLASTIC

ANR-23-CE34-0012 BUBBLPLAST].

**At this time, please address the following queries**

**a)** Please clarify the sources of funding (financial or material support) for your study. List the grants or organizations that supported your study, including funding received from your institution.

We added a Funding section on L455 where we acknowledge our funders: “This study, including the salaries of NY and HM, was supported by ANR-20-CE34–0014 “ATMO-PLASTIC” and ANR-23-CE34-0012 “BUBBLEPLAST” grants from the French Agence Nationale de Recherche. OH was funded by the 80Prime CNRS «4DμPlast» PhD fellowship, and TS by a PhD fellowship from the French ministry of education.”

**b)** State what role the funders took in the study. If the funders had no role in your study, please state: “The funders had no role in study design, data collection and analysis, decision to publish, or preparation of the manuscript.”

**c)** If any authors received a salary from any of your funders, please state which authors and which funders.

**d)** If you did not receive any funding for this study, please state: “The authors received no specific funding for this work.”

Please include your amended statements within your cover letter; we will change the online submission form on your behalf.

**Additional Editor Comments:**

Dear Authors,

I would like you to clarify few things on the manuscript:

1. The height of 1.6-1.7m, can you please justify the selection of this height?

For sampling in the residential environment, the filter holders were placed horizontally at a height of 1.6–1.7 m from the floor, corresponding to the average human inhalation height (Liao et al., 2021). We added clarification to the text on L102–106: “For sampling in the residential environment, the filter holders were placed horizontally at a height of 1.6–1.7 m above the floor in living rooms, corresponding to the average human inhalation height [18]. In bedrooms, a height of 0.5 m was chosen to represent the average inhalation height during sleep, based on the bed height in the studied bedrooms.”

**2.** In cars, how did you standardize the collection of air particles, Was the air-condition always on/off? During the period of collection, were the windows closed or opened? I assume if windows are open and aircorn is on, there will be re-suspension of particles inside the car. How did you standardize that from one car to the other? Same applies inside the house. When sweeping, whether the floor is carpet or tiled, there will also be re-suspension. How was all these accounted for?

Sampling in both cars was carried out while driving the car with the windows closed, AC off, and outside air entering the cabin through vents at a regular rate. In the residences, windows were closed. Some of this was mentioned, but we added further clarification to the Methods section.

**3.** I missed a point on verification of whether it was plastics inhaled, or it is other dust particles? Can you put readers into confidence on how microplastics were verified.

We quantified the concentrations of suspended (floating) MPs in indoor air. The MP identification (verification) was made by Raman microscopy, and example Raman spectra for MP are illustrated in the Supporting Information. The observed MP make up a fraction of total dust particles. We did not quantify the other dust particle concentrations. The estimate of inhaled MP is a calculation that is based on the observed suspended MP and the typical human daily air intake into the lungs.

We have made changes at the end of the introduction to improve flow and clarity on our motivation and objectives.

**Reviewers' comments:**

**Reviewer #1:**

This manuscript is an interesting and important contribution to the study of airborne microplastics. The topic is highly relevant, and the findings add valuable insights into indoor exposure, which makes it even more compelling. The study is well thought out, and the methods are strong.

The comments below are just suggestions to help improve clarity and readability. The research is already solid, and with a few changes, it will be even stronger and easier for readers to engage with.

Thank you for the positive feedback.

**1)** Lines 24–26: The abstract presents interesting findings, but the take-home message could be clearer. Consider emphasizing why your study is particularly significant compared to previous work. For example, rephrase the last two sentences to highlight the critical health implications more directly.

We rephrased the concluding phrase on L24 as follows: “The MP_1–10 µm_ exposure estimates are 100-fold higher than previous estimates that were extrapolated from larger MP sizes, and suggest that the health impacts of MP inhalation may be more substantial than we realize.”

**2)** Line 20: The term "power size distribution law" is mentioned in the abstract but not explained. Since this may not be familiar to all readers, a brief clarification (even a parenthetical note) would improve accessibility.

We added the clarification: “…following a power size distribution law (the number of MP fragments increases exponentially as particle size decreases).”

**3)** Lines 72–78: The inclusion of the car cabin environment is an interesting and novel aspect, but its significance isn’t immediately clear. Would it be useful to briefly explain why car environments were chosen alongside residential space? perhaps highlighting their relevance to daily exposure?

We added a comment early on in the introduction section, on L44, stating that “people spend approximately 5% (Mannan et al., 2021) of their time in a vehicle.”

We also added further motivation to the Materials and Methods section on L89: “Car cabin indoor environment differs from residential indoor environments, such as homes, offices, and commercial buildings and has a different microclimate. Car cabins are smaller, enclosed and mostly made of synthetic materials such as plastic, which can be potential sources of MPs.”

**4)** Lines 62–65: The introduction covers a lot of ground effectively, but the transition to the research gap could be smoother. The sentence starting with "However, despite its potential, only two studies [27,28]..." feels abrupt. Could you introduce this section with a more gradual lead-in?

We tried to follow your advice and reorganized this section.

**5)** Lines 14, 20, 68 .. : The manuscript switches between "MP1–10µm" and "MP1–10 µm" (with and without a space). Standardizing this across the text will improve readability.

Sorry about this. We have checked and corrected the issue throughout the manuscript by choosing the format with space (e.g., MP_1–10 µm_).

**6)** Lines 88–90: The choice of sampling heights (1.6–1.7 m for living rooms and 0.5 m for bedrooms) makes sense intuitively but would benefit from a brief justification. Does this reflect average breathing heights?

The editor made the same comment; we added clarifications on L102-106: “For sampling in the residential environment, the filter holders were placed horizontally at a height of 1.6–1.7 m above the floor in living rooms, corresponding to the average human inhalation height (Liao et al., 2021). In bedrooms, a height of 0.5 m was chosen to represent the average inhalation height during sleep, based on the bed height in the studied bedrooms.”

**7)** Lines 188–192: the author mentions that blank corrections were applied, but it would be helpful to explain if any contamination patterns were observed across different polymer types. Did specific polymers appear more frequently in blanks, and if so, how was this addressed?

We added information on the analysis of blank content to the Results section on L265–269: “A total of five types of polymers were detected in the four blanks, including PE, PP, PET, PHB, and PDMS. Although some polymers, such as PE, PET, and PP, were observed in multiple blanks, the detected MPs varied in both polymer types and size range. Overall, no consistent pattern of contamination was observed among the blanks.”

As mentioned on L205–209: “Polymers found in blanks were subtracted from their corresponding samples based on chemical composition and diameter range (e.g., if the blank presented two particles reported as PE in a [1,2) μm diameter range, these particles were subtracted from the count of PE [1,2) μm class in the sample).

**8)** Lines 320–322: the author reports that MP concentrations were higher in cars than in apartments but also mention that this difference was not statistically significant (p = 0.5). Perhaps rewording to avoid implying a strong contrast would prevent misinterpretation.

We reformulated our phrase to be more clear, L343: “Although the median MP concentration in the car cabin environment was higher than in apartments, this difference was not statistically significant (Mann-Whitney U test; p-value = 0.5) due to the high variability in both environments.”

**9)** Lines 366–369: The estimated inhalation rates are striking, but a brief discussion of uncertainty sources (e.g., variability in human breathing rates, exposure conditions) would help contextualize the robustness of these values.

Breathing rate variability is included in the inhalation uncertainty; exposure conditions (car, office, human movement, surface polymers present, cleaning frequency, ventilation, population) are likely the larger source of variability. We find it premature at this stage to attempt to incorporate exposure variability in the estimate; this is the reason we combine all the exposure variability observed in the literature and in this study into a single broad human exposure estimate. We added context to L398-405**:** “These default values provide a useful reference for harmonized exposure assessment and facilitate consistency across studies. However, actual inhalation rates can vary due to individual physiological differences, activity levels, exposure conditions, and other factors. We multiply these default inhalation rates by the consensus MP concentrations from Table 1, noting that the latter reflect a variety of indoor residential, occupational and car environments. Future studies should refine our understanding of the factors that control indoor MP concentrations, so that exposure estimates can be adapted to a wider spectrum of social and cultural lifestyles.”

**10)** Lines 272–276, 300–301: The figures (1,2,3 and 4) are informative, but are the labels and axis text large enough for clear readability? I suggest improving the image quality by using higher-resolution versions.

It appears that the automatic creation of PDFs on the PLOS One platform after submission reduces the quality of images, making them appear blurry. However, all images were created using high-resolution versions, following the journal’s requirements: TIFF format, less than 10MB in size, and 300–600 dpi. We will try to improve the text size and possibly increase the resolution > 300 dpi…

**11)** Lines 317–319: The discussion highlights how your estimates are higher than previous extrapolations, which is great. However, a sentence explaining why earlier estimates were lower (e.g., due to detection limits, methodology differences) would provide helpful context.

This is slightly confusing. The Line numbers 317-319 are not associated with our text on comparing our new MP concentration estimate with the previous ‘extrapolated’ estimate; the correct old line numbers are 349-352. We added a phrase on new L377 (in blue here): “The new observation-based MP1–10µm concentration estimate of 4,300 MPs/m3 exceeds a previous extrapolated (from µFTIR data > 20 µm) estimate of 36 MPs/m3 by two orders of magnitude but lies within the 95th percentile (19,000 MPs/m3) of that study [37]. This illustrates the difficulty and large uncertainty associated with estimating small MP concentrations based on observations of larger MP by µFTIR.”

**12)** Lines 348–350: The study focuses on indoor exposure, but some readers may wonder how these findings compare to occupational environments with high plastic use (e.g., textile industries). A brief mention of this in the discussion could add depth.

We added the following discussion on L381, citing two influential reviews: “Occupational exposure to airborne MP is pervasive in the textile industry, and multiple diseases have been reported among workers including a threefold increase in lung cancer incidence among nylon flock workers (Landrigan et al., 2023). The precise airborne MP exposure levels are not extensively documented, but have been cited to range from 10,000 to 1,000,000 fibers per m^3^ of air (Wright & Kelly, 2017), which borders the indoor MP concentrations we observe.”

**13)** Lines 381–384: The potential health impacts of inhaling MP1–10µm are mentioned, but could you add one or two specific examples of known biological effects (e.g., inflammation, oxidative stress) to make this more concrete?

We expanded the discussion on L417: “Inhaled MP_1–10µm_ can cross cellular barriers, entering the bloodstream and potentially causing systemic effects, including oxidative stress, immune responses, and even damage to vital organs over time (Revel et al., 2018). Additionally, MPs can carry a range of toxic additives, including heavy metals and persistent organic pollutants, which may exacerbate their harmful effects (Thompson et al., 2009). These chemicals can leach out once inside the body, potentially disrupting endocrine functions (Revel et al., 2018), impairing cellular processes, or increasing the risk of cancer (Prata et al., 2018). The combination of physical and chemical stressors makes the inhalation of microplastics particularly worrisome from a public health perspective.”

**14)** Lines 395–398: The conclusion does a great job summarizing findings, but a forward-looking statement about next steps in this field (e.g., improving detection methods, longitudinal exposure studies) would make for a stronger closing.

Good idea; we added a closing phrase on L442: “Future studies should aim for routine Raman analysis of MP_1–10 µm_ in order to investigate exposure control factors in detail, and include MP inhalation in epidemiological and occupational exposure studies.”

**Reviewer #2:**

1. The study addresses a critical and emerging issue—human exposure to airborne microplastics, particularly in the PM10 range, which is often overlooked.
2. The use of Raman spectroscopy enhances the reliability of microplastic identification and size classification down to 1 µm.
3. The study successfully compares indoor MP concentrations across different environments (residential vs. car cabin), contributing to the understanding of MP inhalation risks.
4. The Introduction effectively highlights the importance of studying airborne microplastics, but a clearer transition into study objectives would improve flow.

Thank you for the encouraging feedback; we have made multiple changes to the introduction to improve logic and flow.

5. The Discussion could be streamlined to avoid redundancy (e.g., repeated explanations about particle inhalation pathways).

We carefully reviewed the Discussion section to improve clarity and reduce redundancy.

6. Figures & Tables are valuable, but better integration into the text with clear references to their significance would be helpful.

We added more references to the Figures and Tables. We acknowledge that PLOS ONE guidelines require figures and their captions to be separated from the main text. To facilitate the readability of the text and take these requirements into account, we have moved all figure captions to a separate section titled “Figure Captions” at the end of the manuscript. At the same time, we have ensured that each figure is clearly referenced within the main text.

7. It would be beneficial to provide more details about sampling duration and how variability in MP concentrations was controlled.

Sampling duration details are now added to Table S1.

8. Clarify whether cross-contamination controls were performed beyond blanks (e.g., during sampling in the field).

To account for cross-contamination throughout all stages from material preparation, sampling on the field, transport, samples storage, and sample preparation and analysis, we did negative controls (so-called blanks). For the blanks, the PTFE filter was placed in the filter holder without pumping. Filter holders for sampling and their corresponding blanks were prepared the same day. The blank filters underwent the same procedure as their corresponding samples. These details are given on L197-201.

9. Some comparisons, such as MP concentrations between apartments and cars, use median values. A statistical significance test (e.g., Mann-Whitney U test) should be explicitly mentioned.

Please see Line 251 in the old version, and Line 343 in the revised MS: “Although the median MP concentration in the car cabin environment was higher than in apartments, this difference was not statistically significant (Mann-Whitney U test; p-value = 0.5) due to the high variability in both environments.”

10. The power law fitting for MP size distribution is informative, but additional explanation about its implications for human exposure would be useful.

We added a clarifying opening phrase to the power law section on L354: “Fragmentation of plastic and MP leads to MP particle size distributions that are characterized by exponentially increasing concentrations of the ever smaller fragments that follow a power law.”

11. The study makes strong claims about increased MP inhalation leading to health risks. While plausible, direct toxicological evidence linking MP1-10µm to specific diseases should be elaborated with relevant citations.

Reviewer 1 requested more details on health risk which we developed on Lines 419-426, including multiple references to relevant papers.

12. Consider discussing how long-term exposure may differ from acute exposure in different environments.

We have looked into the literature on occupational exposure, but a full hour of research online (skimming the most evident hits) does not provide the level of study detail (linking MP concentrations to health effects) that would allow us to discuss this properly.

13. Minor grammatical errors and awkward phrasing should be polished. Example: "Given these findings, and the fact that people spend 90% of their time indoors, the greater potential for exposure to MPs through inhalation in indoor environments should be emphasized." Suggested revision: "Given that people spend 90% of their time indoors, the potential for inhalation exposure to MPs in indoor environments is significantly higher and warrants attention."

Thank you. We have made correction on L43.

14. The study provides valuable insights into indoor airborne microplastics; however, several recent studies on ambient microplastics have been published. I recommend the authors conduct a thorough keyword search to identify and incorporate relevant literature. Strengthening the discussion by comparing indoor and ambient microplastic studies will enhance the manuscript's impact and provide a more comprehensive context for human exposure risks.

Our literature search was done up to early 2024, and we only discuss in detail those papers that provided detailed particle size distributions. Because the funding has ended, and lead postdocs moved on, we have limited resources to include data analyses of papers published since early 2024; sorry for that.

15. Line 35 marine boundary layer it would be good if you will rewrite this sentence as Over the past decade, MPs have been detected in outdoor atmospheric aerosols [5–8] and deposition [9–14], in various parts of the world, from urban and highly industrialized areas [9,13] to remote mountainous regions [10,12], the marine boundary layer [7] and indoor environments. As it will give proper flow For indoor environment cite following references. Unravelling the microplastic contamination: a comprehensive analysis of microplastics in indoor house dust. An in-depth study of dust samples reveals microplastic (MP) contamination in indoor commercial markets.

We updated the text on L37–38 following the recommendation and added the references:

1. Bhat MA. Unravelling the microplastic contamination: A comprehensive analysis of microplastics in indoor house dust. *Indoor and Built Environment*. 2024;33(8):1519-1541. doi:[10.1177/1420326X241248054](https://doi.org/10.1177/1420326X241248054)
2. Bhat, M.A., Gaga, E.O. An in-depth study of dust samples reveals microplastic (MP) contamination in indoor commercial markets. *Euro-Mediterr J Environ Integr* (2025). <https://doi.org/10.1007/s41207-025-00743-z>
16. Lines 62 and 63 check the below articles as Raman has been used in indoor PM microplastics studies. So cite them here as it is contradictory when there is a gap. Airborne microplastic contamination across diverse university indoor environments: A comprehensive ambient analysis. A comprehensive characterization of indoor ambient microplastics in households during the COVID-19 pandemic. A comprehensive characterization of indoor ambient microplastics in households during the COVID-19 pandemic.

We have revised the Introduction section on L66-79: “Several recent studies have successfully applied Raman spectroscopy [29,17,30,31] to assess indoor suspended PM_10_ MPs, providing valuable insights into this emerging field. However, due to variations in methodology approaches, particle size detection limit, and the types of indoor environments studied, further research is essential to build a more comprehensive understanding of the sources, fate and behavior of PM10 MPs in indoor air…”

We have also added the following recommended references:

1. Bhat, M.A. A comprehensive characterization of indoor ambient microplastics in households during the COVID-19 pandemic. *Air Qual Atmos Health* 17, 2017–2033 (2024). <https://doi.org/10.1007/s11869-024-01559-6>
2. Bhat, M.A. Airborne microplastic contamination across diverse university indoor environments: A comprehensive ambient analysis. *Air Qual Atmos Health* 17, 1851–1866 (2024). https://doi.org/10.1007/s11869-024-01548-9

**17.** Line 303 check the missing literature. You can cite the above literature here also.

We have added relevant literature on L324 (in the revised MS) that was previously missing, including the recommended references.

18. Compare your results of lnhaltion rates with the recent studies like. Airborne microplastic contamination across diverse university indoor environments: A comprehensive ambient analysis. A comprehensive characterization of indoor ambient microplastics in households during the COVID-19 pandemic…

While these studies offer valuable insights into the field, they provide particle concentration and overall size distribution, but do not provide detailed information on particle abundance within specific sub-ranges of the full size distribution. To conduct a rigorous comparison with our results using the power law size distribution analysis, such detailed bin-specific data are required. Therefore, we have cited these articles and we acknowledge their relevance, but we cannot provide a rigorous comparison.
