# Supplementary material for: Human exposure to PM10 microplastics in indoor air
Source: PLoS One. 2025 Jul 30;20(7):e0328011. doi: 10.1371/journal.pone.0328011 (PMC12310009; doi:10.1371/journal.pone.0328011)
Supplement: Attachment — Submitted filename: reviewers report.docx [file pone.0328011.s016.docx]

Reviewer’s report:

I think this revised manuscript **’Human exposure to PM_10_ microplastics in indoor air’** is interesting and relevant, and I appreciate the effort the authors have put into addressing the previous comments. Their responses were clear and constructive, and the corresponding changes have improved the overall quality and clarity of the manuscript.

I am satisfied with the revisions and have no further comments. I believe the manuscript is now suitable for publication in its current form.
